# Supplementary material for: Sequential Release of Panax Notoginseng Saponins and Osteopractic Total Flavone from Poly (L-Lactic Acid) Scaffold for Treating Glucocorticoid-Associated Osteonecrosis of Femoral Head
Source: J Funct Biomater. 2023 Jan 4;14(1):31. doi: 10.3390/jfb14010031 (PMC9863477; doi:10.3390/jfb14010031)
Supplement: Supplementary file 1 [file jfb-14-00031-s001.zip › jfb-2084546-supplementary.pdf]

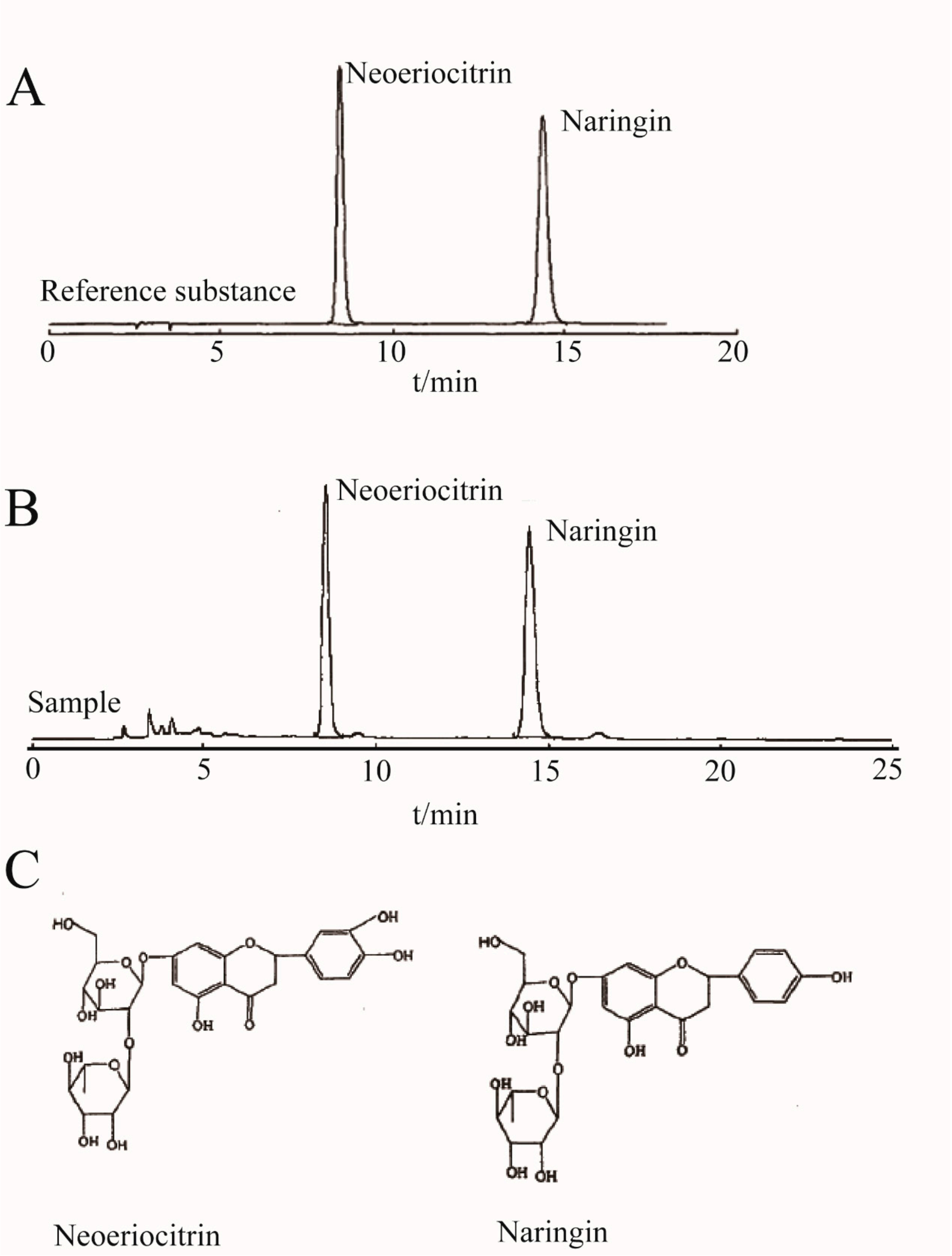

Supplementary Figure S1: Purity identification of osteopractic total flavone (OTF). A, HPLC ion chromatogram of OTF (Reference substance): Neoeriocitrin and Naringin. B, HPLC ion chromatogram of OTF (Sample): Neoeriocitrin and Naringin. C, the molecular formulas of Neoeriocitrin and Naringin.

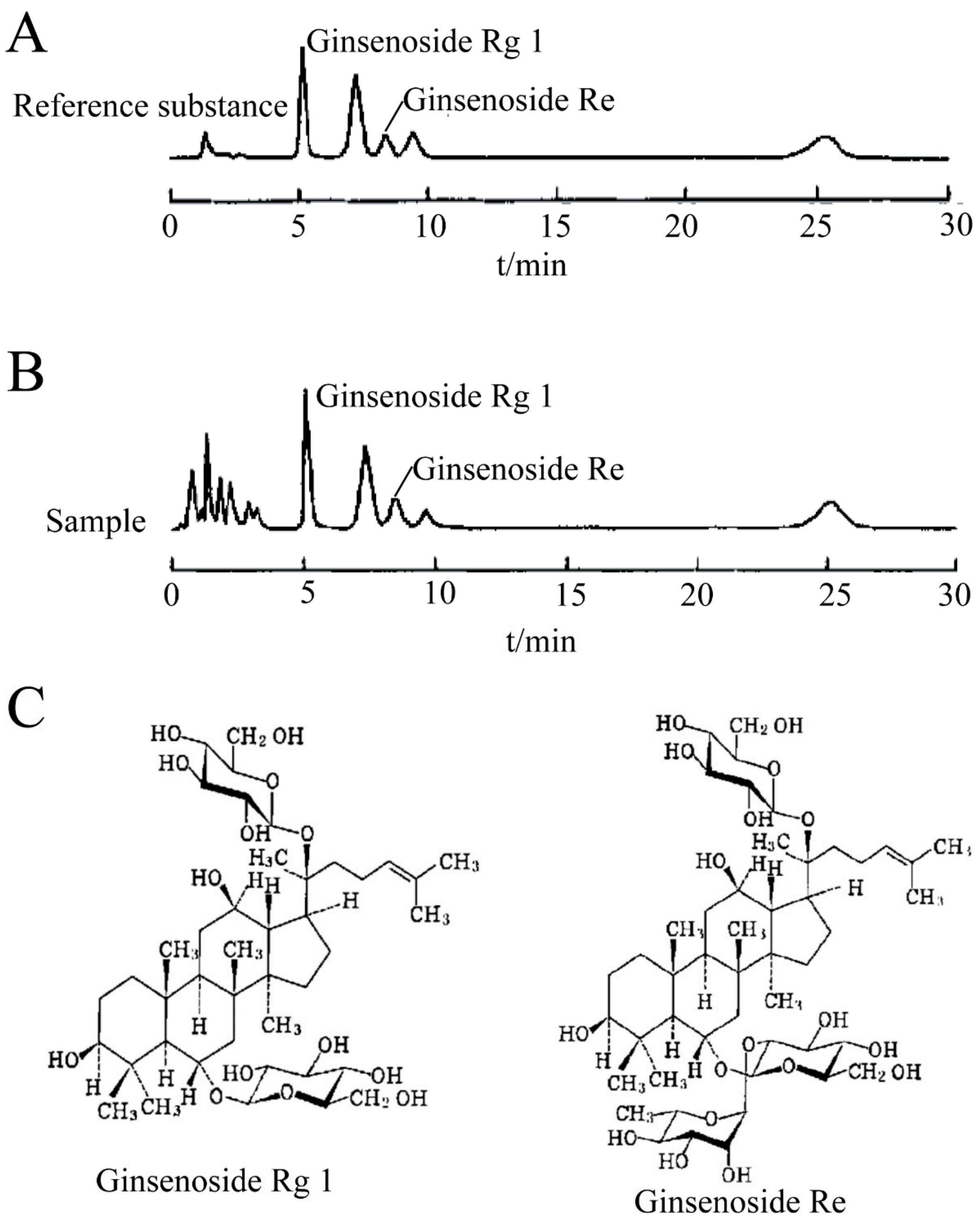

Supplementary Figure S2: Purity identification of panax notoginseng saponins (PNS). A, HPLC ion chromatogram of PNS (Reference substance): Ginsenoside Rg 1 and Ginsenoside Re. B, HPLC ion chromatogram of PNS (Sample): Ginsenoside Rg 1 and Ginsenoside Re. C, the molecular formulas of Ginsenoside Rg 1 and Ginsenoside Re.
